# The Significance of Hypothiocyanite Production via the Pendrin/DUOX/Peroxidase Pathway in the Pathogenesis of Asthma

**DOI:** 10.1155/2017/1054801

**Published:** 2017-11-22

**Authors:** Kenji Izuhara, Shoichi Suzuki, Masahiro Ogawa, Satoshi Nunomura, Yasuhiro Nanri, Yasutaka Mitamura, Tomohito Yoshihara

**Affiliations:** ^1^Division of Medical Biochemistry, Department of Biomolecular Sciences, Saga Medical School, Saga, Japan; ^2^Infectious Disease Unit, Asia International Institute of Infectious Disease Control, Teikyo University, Tokyo, Japan

## Abstract

Inhaled corticosteroids (ICSs) are used as first-line drugs for asthma, and various novel antiasthma drugs targeting type 2 immune mediators are now under development. However, molecularly targeted drugs are expensive, creating an economic burden on patients. We and others previously found pendrin/SLC26A4 as a downstream molecule of IL-13, a signature type 2 cytokine critical for asthma, and showed its significance in the pathogenesis of asthma using model mice. However, the molecular mechanism of how pendrin causes airway inflammation remained elusive. We have recently demonstrated that hypothiocyanite (OSCN^−^) produced by the pendrin/DUOX/peroxidase pathway has the potential to cause airway inflammation. Pendrin transports thiocyanate (SCN^−^) into pulmonary lumens at the apical side. Peroxidases catalyze SCN^−^ and H_2_O_2_ generated by DUOX into OSCN^−^. Low doses of OSCN^−^ activate NF-*κ*B in airway epithelial cells, whereas OSCN^−^ in high doses causes necrosis of the cells, inducing the release of IL-33 and accelerating inflammation. OSCN^−^ production is augmented in asthma model mice and possibly in some asthma patients. Heme peroxidase inhibitors, widely used as antithyroid agents, diminish asthma-like phenotypes in mice, indicating the significance of this pathway. These findings suggest the possibility of repositioning antithyroid agents as antiasthma drugs.

## 1. Introduction

Asthma is a common and chronic respiratory disease characterized by variable symptoms and features―wheezing, shortness of breath, cough, and expiratory airflow limitation [1]. Asthma is estimated to affect at least 300 million people worldwide, making it a significant medical and social problem. Inhaled corticosteroids (ICSs) are used as first-line drugs for asthma. Although ICSs are very effective, 5–10% of asthma patients are estimated as having severe asthma characterized by difficulty to achieve disease control despite high-dose ICSs plus long-acting *β*2-agonists or oral corticosteroids, which accounts for about 50% of the total costs for treating asthma [2, 3]. It is known that type 2 inflammation is dominant in the pathogenesis of asthma [4]. Based on this immunological background, various novel antiasthma drugs targeting type 2 immune mediators―interleukin- (IL-) 4, IL-5, IL-13, TSLP, IL-33, and CRTH2―are now under development [5]. However, molecularly targeted drugs, mostly biologics, are expensive, creating an economic burden on patients. Therefore, it is of great importance to elucidate the pathogenesis of severe asthma to help identify therapeutic strategies that will be more affordable for these patients.

It has been sporadically reported that IL-4 and/or IL-13, signature type 2 cytokines, influence anion transport in airway tissues [6]. However, the underlying mechanism of how anion transport in airway tissues leads to inflammation has not been sufficiently explained. We and others previously found that pendrin/SLC26A4, an anion transporter located at the apical side of airway epithelial cells, is a downstream molecule of the IL-4/IL-13 signals that plays an important role in the pathogenesis of asthma [7, 8]. We then investigated how pendrin causes airway inflammation, pinpointing the significance of the hypothiocyanite (OSCN^−^) production via the pendrin/DUOX/peroxidase pathway [9, 10]. These results have revealed for the first time the involvement of anion or its derivative in the pathogenesis of asthma. Moreover, these findings suggest to us that we can apply antithyroid agents, pan-heme peroxidase inhibitors, to drug repositioning for antiasthma drugs.

In this article, we describe how we started our research and how we have arrived at these findings.

## 2. Discovery of Pendrin as a Downstream Molecule of the IL-4/IL-13 Signals

IL-4 and IL-13 are signature cytokines of type 2 inflammation produced by T_H_2 cells, follicular helper T cells, group 2 innate lymphoid cells (ILC2), eosinophils, mast cells, and basophils [11–14]. A number of analyses using asthma model mice have established the significance of IL-4 and/or IL-13, particularly the latter, in the pathogenesis of asthma [11, 15–17]. Based on these findings, several IL-4 or IL-13 signaling antagonists such as tralokinumab and dupilumab are now under clinical development as antiasthma drugs [18, 19]. To identify a novel mediator involved in asthma pathogenesis downstream of the IL-13 signals, we and others previously used DNA microarray to search for IL-13-induced molecules in human airway epithelial cells, finding that the *SLC26A4* gene encoding pendrin is a downstream molecule of IL-13 [7, 20, 21]. Moreover, Pedemonte et al. found that IL-4 increases thiocyanate (SCN^−^) transport in human airway epithelial cells independently of the cystic fibrosis transmembrane conductance regulator (CFTR) [22]. They found that among the investigated transporters, the *SLC26A4* gene was significantly induced by IL-4 and that pendrin is responsible for the SCN^−^/Cl^−^ exchange. Thus, pendrin appears to be an IL-4- or IL-13-inducible molecule.

In agreement with the *in vitro* experiments, we and others have demonstrated that pendrin is highly expressed in the lungs of asthma model mice such as ovalbumin-inhaled, IL-13-inhaled, and IL-13 transgenic mice [7, 20–23]. We showed that pendrin is expressed in the apical side of airway epithelial cells in ovalbumin-inhaled mice [7]. Nonciliated airway epithelial cells are likely the main pendrin-expressing cells when stimulated by IL-4/IL-13, because pendrin expression is upregulated in the IL-13-overexpressing mice, in which STAT6 is expressed only in nonciliated airway epithelial cells [21]. Moreover, pendrin expression was enhanced in model mice of both acute and chronic asthma [23].

Since the *SLC26A4* gene is an IL-4/IL-13-inducible molecule, it was reasonable to think that STAT6, a transcriptional factor critical for the IL-4/IL-13 signals, regulates the expression of the *SLC26A4* gene. Nofziger et al. found that there exist two consensus binding sites for STAT6 (TTC(N_4_)GAA) at −3472 to −3463 (motif 1) and −1812 to −1803 (motif 2) of the 5′-flanking region of the *SLC26A4* gene [24, 25]. Vanoni et al. showed that although both consensus sequences can bind STAT6 following IL-4 exposure, IL-4- or IL-13-inducible pendrin expression requires only motif 2 [25]. These results suggest that IL-4 or IL-13 induces expression of the *SLC26A4* gene in a *cis*-regulating manner.

It has been thereafter demonstrated that in addition to IL-4 and IL-13, pendrin expression in lung tissues or airway epithelial cells can have other causes. These include various cytokines, such as IL-1*β* [22, 26] and IL-17A [27–29]. Also possible are various environmental stimuli such as silica [30], welding fumes [31], C_60_ fullerene [32], and single-wall carbon nanotubes [33]. In addition, pathogenic microbes or microbe-derived molecules―pertussis toxin [27, 34] and a combination of interferon-*γ* (IFN-*γ*) and rhinovirus [8]―can be a cause. These findings expand the potential of pathophysiological roles of pendrin. It is of note that the combination of IL-13 and IL-17A enhances pendrin expression in airway epithelial cells [29]. Since expression of IL-17A is a hallmark of severe asthma correlated with infiltration of neutrophils [35], pendrin may be maximally expressed in severe asthma patients.

## 3. The Pathological Roles of Pendrin in Asthma

Using model mice, we and Nakagami et al. have previously demonstrated the significance of pendrin in the pathogenesis of airway allergic inflammation [7, 8]. Overexpression of pendrin in bronchial tissues leads to mucus hyperproduction, enhanced airway hyperreactivity (AHR), and upregulation of chemokine expression followed by infiltration of neutrophils [7]. Reciprocally, ovalbumin challenge for pendrin-deficient mice decreases airway reactivity and infiltration of inflammatory cells, including eosinophils in bronchoalveolar lavage fluid (BALF), although systemic IgE production, mucus production, and the production of type 2 cytokines do not change [8]. Accordingly, it has been reported that pendrin expression was enhanced in asthma patients compared to control subjects [36], although there is a contradictory report [21]. Moreover, it has been shown that pendrin is highly expressed in the nasal mucosa of patients with chronic rhinosinusitis with nasal polyps (CRSwNP) and allergic rhinitis (AR) [29, 37]. It is known that the existence of eosinophilic CRS in asthma patients is a risk factor for worsening asthma and that the presence of concomitant AR can affect the severity of asthma [38]. These results support the pathogenic significance of pendrin in both upper and lower respiratory allergic inflammation.

The involvement of pendrin in the pathogenesis of airway inflammation has been expanded into COPD and pertussis disease. We found that pendrin expression was enhanced in the lung tissues of esterase-inhaled mice mimicking COPD [7]. In COPD model mice, pendrin is expressed at the apical side of epithelial cells, as it is in asthma model mice, followed by expression of Muc5ac and Muc5b. Since it is known that expression of IL-17A and IL-13 is enhanced in COPD patients [39–41], the combination of these cytokines may induce pendrin expression in COPD. Scanlon et al., moreover, have demonstrated that *Bordetella pertussis* induces pendrin expression in an IL-17A-dependent manner and that pendrin deficiency improves *Bordetella pertussis*-induced inflammation but does not affect bacteria colonization [27].

## 4. The Role of Pendrin in Airway Surface Liquid (ASL)

Airway surfaces are covered by a thin layer of fluid, ASL, whose composition and volume are critical to ensure proper mucociliary clearance and maintain innate defense systems [6]. The amount and composition of ASL are regulated by the balance between fluid secretion and absorption coordinated by several ion channels and transporters, including pendrin. Nakagami et al. and Lee et al. showed that ASL is thickened in pendrin-deficient tracheal cells stimulated by IL-13 and deaf patients carrying pendrin mutations, probably because of dysregulated anion transport [8, 42]. This suggests the possibility that thickened ASL can enhance mucus clearance and improve airway function, which may at least partially explain how periostin deficiency improves asthma-like phenotypes. Increased pendrin expression following allergen challenges may lead to ASL dehydration and then to airway inflammation and obstruction, thereby exacerbating asthma.

## 5. The Pathological Role of the Pendrin/DUOX/Peroxidase Pathway in Asthma

The finding that pendrin is important for the onset of airway inflammation suggested to us that anions transported by pendrin, or their derivatives, could play an important role in asthma.

**Figure 1:**
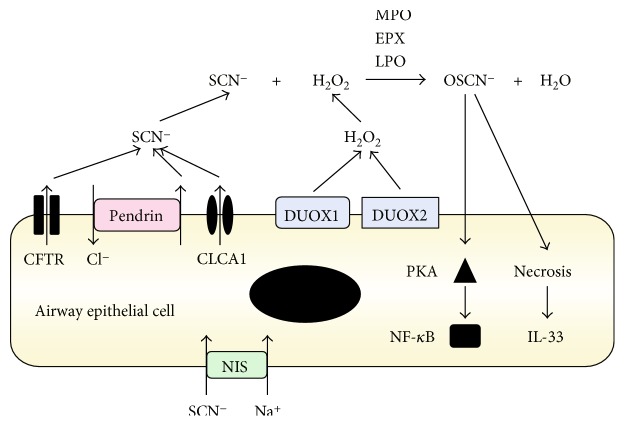
Schematic model of OSCN^−^ production via the pendrin/DUOX/peroxidase pathway in airway epithelial cells (modified from [9]). In airway epithelial cells, SCN^−^ is actively transported into pulmonary lumens via NIS/SLC5A5 at the basal side and via several anion transporters including CFTR and pendrin/SLC26A4 at the apical side. SCN^−^ together with H_2_O_2_ generated by Duox1 and Duox2 is catalyzed by peroxidases into OSCN^−^. Three peroxidases including MPO, EPX, and LPO are involved in this reaction. A low dose of OSCN^−^ activates NF-*κ*B via PKA, whereas a high dose of OSCN^−^ causes necrosis followed by release of IL-33 in airway epithelial cells. It is of note that if peroxidases are inhibited, it would protect airway epithelial cells against inflammation.

Among various anions, we focused on SCN^−^, because pendrin can transport SCN^−^ into the apical side of airway epithelial cells [22] and OSCN^−^ derived from SCN^−^ plays a critical role in the innate defense of mucosal surfaces [43–45]. SCN^−^ is incorporated from the basal side into airway epithelial cells by the Na^+^I^−^ symporter (NIS)/SLC5A5 and then is actively transported into pulmonary lumens at the apical side by CFTR and pendrin (Figure 1). In contrast, DUOX1 and/or DUOX2, members of the NOX/DUOX family, generate hydrogen peroxide (H_2_O_2_) in pulmonary lumens. SCN^−^ and H_2_O_2_ are catalyzed into OSCN^−^ by three peroxidases—myeloperoxidase (MPO), eosinophil peroxidase (EPX), and lactoperoxidase (LPO)—expressed in neutrophils, eosinophils, and epithelial cells, respectively, in the lung tissue. OSCN^−^ has potent antimicrobial properties against bacteria, viruses, and fungi, as seen in cystic fibrosis patients, whose susceptibility to chronic respiratory infections increases in proportion to impaired CFTR function [46].

**Figure 2:**
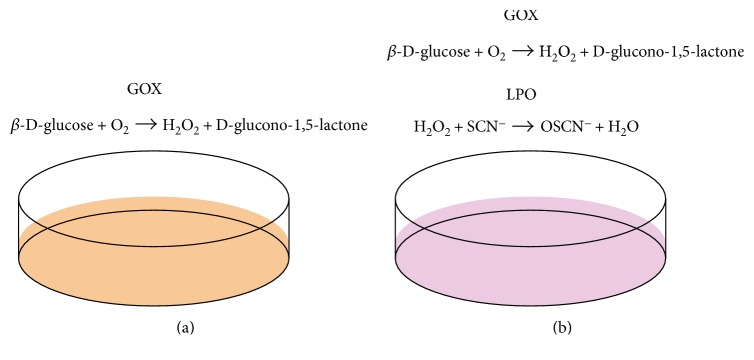
*In vitro* OSCN^−^ production system (modified from [9]). When only *β*-D-glucose and glucose oxidase (GOX) are added into the system using airway epithelial cells (H292 cells), H_2_O_2_ is generated (a). When more SCN^−^ and LPO are added, H_2_O_2_ is catalyzed into OSCN^−^ (b). Thus, in this system, the oxidative activities of H_2_O_2_ and OSCN^−^ can be estimated with or without addition of SCN^−^ and LPO.

We examined whether the production of OSCN^−^ leads to inflammation in airway epithelial cells (H292 cells) using an *in vitro* OSCN^−^ production system [9]. In this system, when we added only *β*-D-glucose and glucose oxidase (GOX) in the reaction mixture, H_2_O_2_ was generated by GOX using *β*-D-glucose and oxygen (Figure 2(a)). When SCN^−^ and LPO were furthermore added in the mixture, OSCN^−^ was generated by LPO via the oxidation of SCN^−^ with H_2_O_2_ (Figure 2(b)). Using this system, we compared the ability of H_2_O_2_ and OSCN^−^ to activate NF-*κ*B, finding that OSCN^−^, but not H_2_O_2_, activated NF-*κ*B (Figure 1). Activation of NF-*κ*B in airway epithelial cells is important for production of chemokines and inflammatory cytokines as well as for expression of adhesion molecules accelerating type 2 immunity [47]. OSCN^−^ was sensed by protein kinase A (PKA) followed by the dimerization of PKA. The regulatory subunit of type I PKA is dimerized through a disulfide bond followed by increased affinity for the substrates, demonstrating that oxidative stress is changed to intracellular signaling through PKA, independently of cAMP [48]. The stronger oxidative ability of OSCN^−^ compared to H_2_O_2_ may be due to the existence of a detoxifying system for H_2_O_2_, mainly by catalase in airway epithelial cells. Furthermore, OSCN^−^ in high doses caused necrosis of the cells, inducing release of IL-33, which acts on several immune cells—ILC2, mast cells, basophils, eosinophils, and T_H_2 cells accelerating type 2 inflammation [49]. To our knowledge, OSCN^−^ is the first anion to activate NF-*κ*B in epithelial cells. Thus, we have shown that OSCN^−^ produced by the pendrin/DUOX/peroxidase pathway potentially plays an important role in the pathogenesis of asthma.

## 6. Enhancement of the OSCN^−^ Production System in Asthma

We next examined whether OSCN^−^ production via the pendrin/DUOX/peroxidase pathway is enhanced in asthma model mice (Table 1) [10]. Expression of pendrin was significantly enhanced, as shown in a previous study [7], whereas expression of CFTR did not change. Expression of all three heme peroxidases (*Mpo*, *Epx*, and *Lpo*) together with peroxidase activities in BALFs was enhanced in allergen-challenged mice. Moreover, the expression of DUOX1, but not DUOX2, was also significantly enhanced in allergen-challenged mice, consistent with a previous report showing that IL-4 can induce DUOX1 [50]. These results suggest that the OSCN^−^ production machinery is enhanced in asthma model mice. We then investigated whether expression of the heme peroxidases was enhanced in the bronchial tissues of mild to moderate asthma patients well controlled with inhaled corticosteroids [50]. The peroxidase activities and the expression levels of *LPO* were not statistically enhanced. However, some patients showed distinctly high peroxidase activities and *LPO* expression. The clinical severity of their asthma, modified treatment for these patients, and/or heterogeneity among asthma patients may affect airway peroxidase expression, although the precise factor causing the difference is unclear at this moment. Expression of neither *EPO* nor *MPO* was detected. These results confirm that OSCN^−^ production via pendrin/DUOX/peroxidase is augmented in asthma model mice and possibly in some asthma patients.

To define the pathological roles of peroxidase in bronchial asthma, we applied heme peroxidase inhibitors to an asthma mouse model [10]. We examined the effects of 2-mercapto-1-methylimidazole (methimazole) and 6-propyl-2-thiouracil (PTU), which are agents that inhibit all peroxidases and are widely used as antithyroid agents targeting thyroid peroxidase. The long administration of methimazole (orally every day from the start of sensitization (day 0)) completely inhibited airway inflammation―enhanced AHR, infiltration of inflammatory cells in BALF, and histological changes (Table 2). Short administration (from two days before the start of the allergen airway challenge (day 20)) inhibited inflammation less so, yet significantly. Another peroxidase-inhibiting antithyroid agent, PTU, showed effects similar to but weaker than those of methimazole. These results strongly suggest that heme peroxidase activities are critical for the onset of allergic airway inflammation in these model mice. Our results appear consistent with the findings of several reports showing that accidental administration of antithyroid agents provided beneficial effects to asthma patients [51, 52], although there is one conflicting report [53]. It is of note that in most patients, bronchial asthma was exacerbated by discontinuing or tapering off antithyroid agents [51, 52].

Next, we examined which peroxidase dominantly contributes to the onset of allergic airway inflammation using mice deficient in each of the three peroxidases (*Mpo*, *Epx*, and *Lpo*) [10]. *Epx*- and *Lpo*-deficient mice showed a nominal but not statistically significant decrease of AHR compared to their control littermates, whereas the *Mpo*-deficient mice showed no change of AHR (Table 2). Furthermore, infiltration of eosinophils and T cells was decreased in the BALF of the *Lpo*-deficient mice, whereas there was no change in infiltration in the *Mpo*- or *Epx*-deficient mice. These results suggest that the contributions of the three peroxidases are redundant in the onset of allergic airway inflammation. However, *Lpo* appears to be dominant among the peroxidases.

Taking these results together, we assume that whereas the OSCN^−^ production system may be an innate host defense mechanism in the lung, this misplaced production of OSCN^−^ is likely to contribute to pulmonary inflammation, causing deleterious effects in response to airway allergen provocation.

## 7. Clinical Application of the Pathological Significance of the Pendrin/DUOX/Peroxidase Pathway to Asthma

The findings showing the importance of the OSCN^−^ production via the pendrin/DUOX/peroxidase pathway in asthma indicate that all of the machineries of the OSCN^−^ production system can be viewed as potential novel therapeutic targets for asthma. It is of note that we have confirmed that heme peroxidase inhibitors widely used as antithyroid agents are efficacious for inhibiting allergic airway inflammation in mice. This suggests that we can apply antithyroid agents to drug repositioning for antiasthma drugs. Drug repositioning, which is the process of finding new therapeutic indications for existing drugs, is now seen as a less expensive alternative to drug discovery and development [54, 55]. The use of antithyroid agents could be the first example of drug repositioning for asthma. Various antiasthma drugs targeting type 2 immune mediators, such as IL-4, IL-5, IL-13, TSLP, IL-33, and CRTH2, are now under development [5]. However, to develop novel drugs, particularly biologics, huge investments of time and money are required, and safety risks are involved. Moreover, most molecularly targeted drugs for asthma under development are biologics, which are relatively expensive. Clearly, drug repositioning in asthma can potentially decrease the economic burden on asthma patients.

Moreover, the importance of the OSCN^−^ by the pendrin/DUOX/peroxidase pathway in asthma can be applied to airway inflammation in smokers. Plasma SCN^−^ levels in smokers are almost three times higher than in nonsmokers (130–140 *μ*M versus 40–50 *μ*M) [56, 57]. It is well known that smoking is associated with poor control, decrease of lung function, and enhanced corticosteroid resistance in asthma [58, 59]. However, no deleterious effects of SCN^−^ or OSCN^−^ derived from tobacco on the lungs have yet been reported. The findings suggest the possible involvement of the OSCN^−^ production system in how smoking affects asthma or other smoking-related pulmonary diseases, giving us clues on how best to treat asthma patients who smoke.

## 8. Conclusion

After showing that pendrin/SLC26A4 is a downstream molecule of IL-13 and that it is actively involved in the pathogenesis of airway allergic inflammation, we investigated the underlying molecular mechanism of how this occurs. As a result, we have demonstrated the significance of OSCN^−^ production via the pendrin/DUOX/peroxidase pathway in allergic airway inflammation. The most important clinical point of this finding is that it suggests the possibility of using antithyroid agents, pan-heme peroxidase inhibitors, as repositioned antiasthma drugs. If we can apply this strategy to asthma patients, it should greatly reduce the cost of treating asthma.

## Figures and Tables

**Table 1:** Change of the machineries of the OSCN^−^ production system in asthma model mice and asthma patients.

Molecule	Asthma model mouse	Asthma patient
Pendrin	**↑**	**↑**/→^∗^
CFTR	→	
Heme peroxidase		
Myeloperoxidase	**↑**	ND
Eosinophil peroxidase	**↑**	ND
Lactoperoxidase	**↑**	**↑**/→
DUOX1	**↑**	→
DUOX2	→	**↑**/→

Expression changes of pendrin, CFTR, MPO, EPX, LPO, DUOX1, and DUOX2 in asthma model mice and asthma patients are depicted. ND: not detected. ^∗^Referred from [9, 10].

**Table 2:** Effects of peroxidase inhibitor and genetic deficiency of each peroxidase on asthma model mice (referred from [10]).

Phenotype	Met-L	Met-S	*Lpo* ^−^	*Epx* ^−^	*Mpo* ^−^
AHR	↓↓↓	↓↓	↓	↓	→
BALF					
Eosinophil	↓↓↓	↓↓	↓↓	→	→
T cell	↓↓↓	↓	↓↓	↓	→
Neutrophil	↓↓↓	↓↓	↓	→	→
Macrophage	→	→	↓	→	→

Effects of the long (Met-L) or short (Met-S) administration of methimazole or genetic deficiency of *Lpo* (*Lpo*^−^), *Epx* (*Epx*^−^), and *Mpo* (*Mpo*^−^) on enhanced AHR and the numbers of eosinophils, T cells, neutrophils, and macrophages in BALF of asthma model mice are depicted.
